# Cisplatin remodels the tumor immune microenvironment via the transcription factor EB in ovarian cancer

**DOI:** 10.1038/s41420-021-00519-8

**Published:** 2021-06-05

**Authors:** Wei Liu, Yanqiu Wang, Yunkai Xie, Tianyu Dai, Mingjun Fan, Changzhong Li, Yonghui Zou

**Affiliations:** 1 Department of Gynecology, Shandong Provincial Hospital, Cheeloo College of Medicine, Shandong University, 250021 Jinan, Shandong China; 2 Department of Operating Room, Shandong Provincial Hospital Affiliated to Shandong First Medical University, 250021 Jinan, Shandong China; 3 Department of Gynecology, Shandong Provincial Hospital Affiliated to Shandong First Medical University, 250021 Jinan, Shandong China

**Keywords:** Cancer microenvironment, Cancer microenvironment

## Abstract

The mortality rate of ovarian cancer (OC) remains the highest among all gynecological malignancies. Platinum-based chemotherapies are effective in treating most OC cases. However, chemoresistance is still a major challenge for successful OC treatments. Emerging evidence has highlighted that the modulation of the tumor immune microenvironment is involved in chemoresistance, but the mechanism remains unclear. This study aimed to investigate whether resistance to cisplatin (CDDP), the standard treatment for OC, is due to the remodeling of the tumor immune microenvironment by the transcription factor EB (TFEB). We hypothesized that TFEB is not essential for tumor survival but is associated with CDDP resistance. We collected 20 tissue samples of OC patients who had not undergone chemotherapy or radiotherapy prior to surgery. We cultured OC cell lines and performed cell transfection and assays as well as analytical, fluorescence microscopy, and immunohistochemical techniques to explore a novel function of TFEB in remodeling the tumor immune microenvironment in OC. We found a positive correlation between TFEB and programmed cell death-ligand 1 (PD-L1), PD-L2, and HLA-A expression in OC cells and tissues. We also found that CDDP treatment induced TFEB nuclear translocation, thus increasing PD-L1 and PD-L2 expression to foster an immunosuppressive tumor microenvironment, which mediates tumor immune evasion and drug resistance. Interestingly, TFEB also regulated HLA-A expression, which increases the tumor immunogenicity of OC. Finally, in a syngenic murine model of OC, we observed the therapeutic benefit of CDDP plus programmed cell death-1 (PD-1) inhibitor, which enhanced the cytolytic activity of CD8^+^ T cells and inhibited tumor growth. Our study illustrates the important role of TFEB in regulating the tumor immune microenvironment in OC.

## Introduction

Ovarian cancer (OC) is still one of the most common and fatal gynecological malignancies worldwide^1^. Triweekly intravenous treatment with paclitaxel and carboplatin with or without bevacizumab is the most widely accepted standard of care for OC patients. Paclitaxel- and platinum-based chemotherapies are effective in treating most OC cases. However, over 70% of patients experience recurrence and eventually develop chemoresistance^2^.

Recent studies have highlighted that the tumor immunosuppressive microenvironment is an important driver in mediating chemoresistance^3,4^. Tumor cells can escape the host immune attack by enhancing the expression of immune checkpoints, such as programmed cell death-ligand 1 (PD-L1) and programmed cell death-1 (PD-1). The expression of these immune checkpoints can also be induced by treatment with many chemotherapeutic agents^5,6^. The emergence of immune checkpoint inhibitors (ICIs) and/or innovative combinations of chemotherapeutic agents with ICIs has become a clinically effective treatment modality for various cancers (melanoma, non-small cell lung cancer, renal cancer, and bladder cancer)^7–9^. Although there is also a solid rationale for using ICIs in OC patients, the clinical data presented to date are not very convincing^10–14^. Therefore, further research on OC treatment with ICIs or innovative combinations is necessary.

The transcription factor EB (TFEB), a member of the MiT/TFE family, has recently emerged as a master regulator of lysosomal biogenesis, cellular energy homeostasis, and immune responses^15–17^. In multiple types of human cancers, the expression and activity of TFEB are elevated and are associated with enhanced motility and proliferation; thus, it was originally described as an oncogene^18–21^. TFEB is also involved in chemoresistance via lysosomal biogenesis and lysosomal drug sequestration^22–25^. Recently, Zhang et al.^26^ reported that TFEB induces PD-L1 to mediate renal cell carcinoma (RCC) immune evasion and resistance to mTOR inhibition. Therefore, this study aimed to investigate whether TFEB is involved in cisplatin (CDDP) resistance by regulating the tumor immune microenvironment in OC.

## Results

### TFEB does not affect proliferation of OC cells

**Fig. 1:**
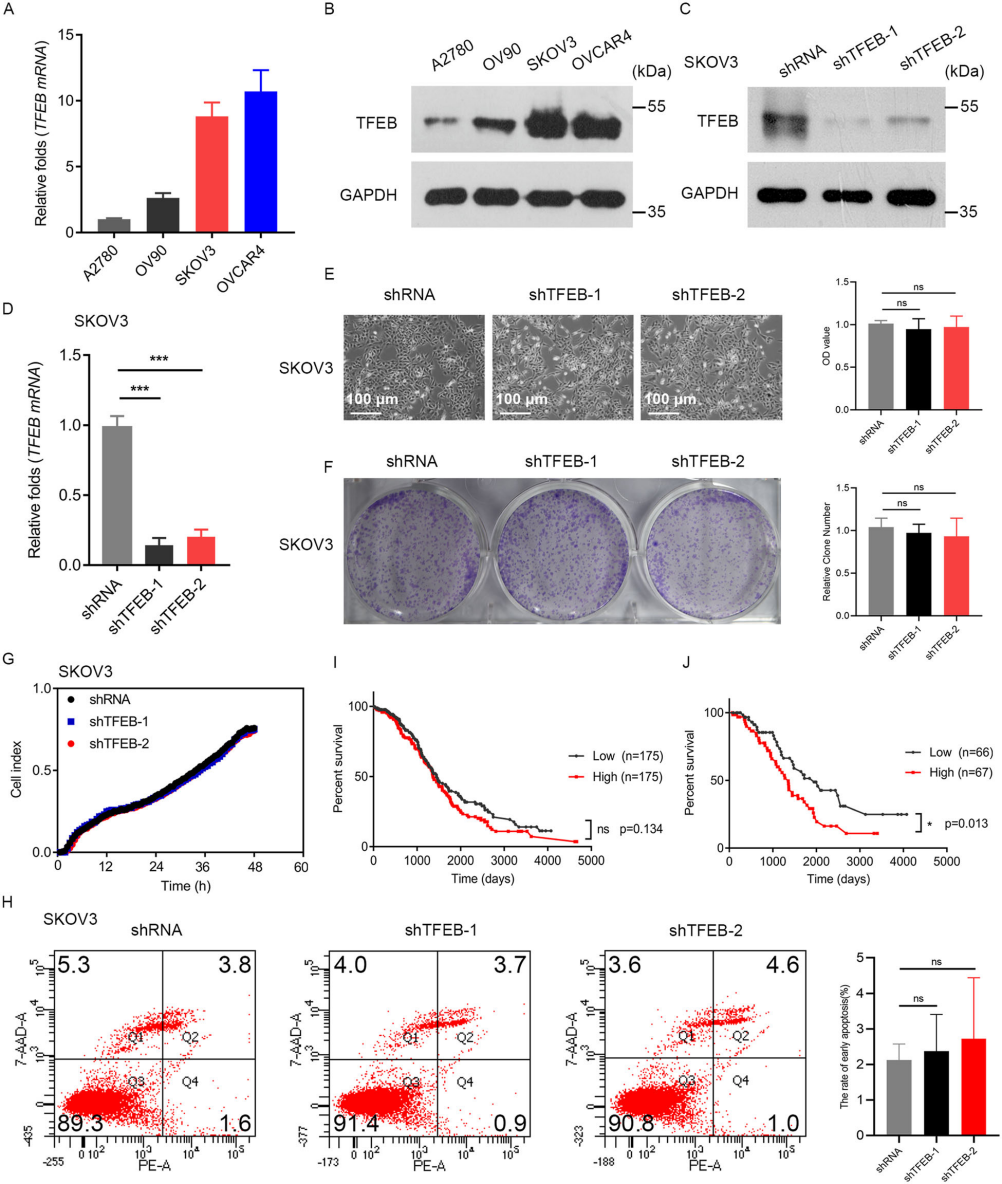
TFEB does not affect proliferation of OC cells. **A** Expression of TFEB in OC cell lines was detected by real-time quantitative reverse transcription polymerase chain reaction (qRT-PCR). **B** Expression of TFEB in OC cell lines was detected by western blotting. **C** shRNA knockdown of TFEB was analyzed by western blotting. **D** shRNA knockdown of TFEB was analyzed by qRT-PCR. **E** Cell proliferation was analyzed by morphology following the knockdown of TFEB (magnification ×400). Scale bars = 100 μm. The corresponding OD values detected at 450 nm were shown. **F** Colony-forming assay of SKOV3 cells following the knockdown of TFEB. Representative histograms were shown. **G** Cell viability was analyzed using an xCELLigence RTCADP instrument following the knockdown of TFEB. **H** Cell apoptosis of SKOV3 cells was determined by flow cytometry. **I** Survival curve of TFEB expression and patient prognosis in all OC patients from The Cancer Genome Atlas (TCGA) database. **J** Survival curve of TFEB expression and patient prognosis in the cisplatin treatment group from the TCGA database. High high-level TFEB group, low low-level TFEB group. Data are expressed as mean ± standard deviation. *p < 0.05; ***p < 0.001; ns not significant.

TFEB levels are elevated in different human cancers and are associated with occurrence and poor prognosis. To clarify the role of TFEB in OC, we first compared TFEB expression in a panel of human OC cell lines. SKOV3 cells showed the highest expression of TFEB, followed by OVCAR4, OV90, and A2780 cells (Fig. 1A, B). TFEB was knocked down in SKOV3 cells (Fig. 1C, D). The result showed that downregulation of TFEB does not affect the proliferation of SKOV3 cells (Fig. 1E–G). These results were further validated with an apoptosis assay (Fig. 1H). Additionally, TFEB was knocked down in OVCAR4 cells and overexpressed in A2780 cells, and it did not affect the proliferation of cells (Supplementary Fig. 1A–D). To validate the role of TFEB in the efficacy of anticancer therapy, we also analyzed publicly available data from The Cancer Genome Atlas (TCGA) database on TFEB expression in patients. As shown in Fig. 1I, no significant correlation existed between TFEB expression and patient prognosis in all OC patients. However, in the CDDP treatment group, TFEB expression was negatively correlated with patient prognosis (Fig. 1J). This finding indicates that TFEB may be a good drug-resistant marker but not a potent tumor promoter in specific tumor types such as OC.

### Therapy-activated TFEB acts as a critical regulator to control lysosomal biogenesis and drug resistance

**Fig. 2:**
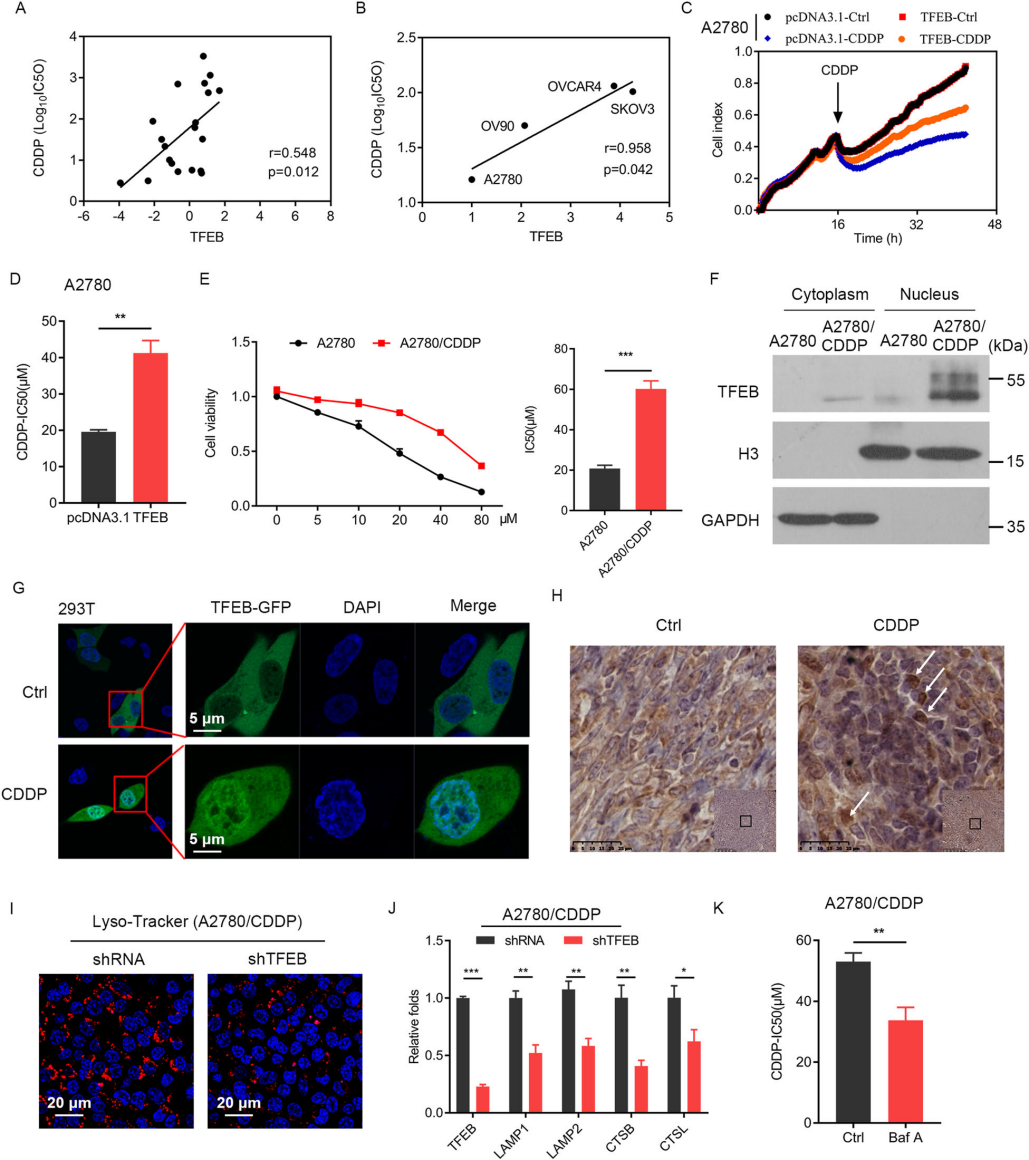
Therapy-activated TFEB acts as a critical regulator to control lysosomal biogenesis and drug resistance. **A** Correlation analysis of the TFEB expression level and OC cells sensitivity to cisplatin (CDDP) from the Genomics of Drug Sensitivity in Cancer (GDSC) database. **B** Correlation analysis of TFEB expression in a panel of human cell lines (A2780, OV90, SKOV3, and OVCAR4 cells) and their sensitivity to CDDP from GDSC database. **C** Cell proliferation curve of A2780 cells. A2780 cells were transduced with an empty vector plasmid (pcDNA3.1) or a pcDNA3.1-TFEB plasmid (TFEB) and were treated with or without CDDP (10 µM). **D** Tolerance of A2780 cells to CDDP, as determined by CCK-8 assay. A2780 cells were transduced with an empty vector plasmid (pcDNA3.1) or a pcDNA3.1-TFEB plasmid (TFEB). **E** Cell viability of A2780 cells and resistant cells (A2780-CDDP) after were treated with cisplatin at corresponding concentrations for 48 h was detected by CCK-8 assay. Half maximal inhibitory concentration (IC50) of CDDP was calculated. **F** TFEB activity in A2780 and A2780-CDDP cells, as determined by western blotting. **G** The intracellular localization of TFEB in 293T cells was determined by a confocal microscope. 293T cells were transduced with TFEB-GFP plasmid and then treated with vehicle or CDDP (10 µM) for 12 h. Scale bars = 5 μm. **H** The effects of CDDP treatment on TFEB expression in the vivo mouse tumors were visualized by immunohistochemistry (arrows indicate nuclear staining). Scale bars = 25 μm. **I**, **J** Effects of TFEB depletion on lysosome biogenesis and their associated gene expression in resistant cells. Scale bars = 20 μm. **K** Bafilomycin A1 (Baf A) increased the sensitivity of resistant cells to CDDP. Data are expressed as mean ± standard deviation. *p < 0.05; **p < 0.01; ***p < 0.001.

We next explored whether TFEB-driven changes contributed to CDDP resistance. We first analyzed the correlation between TFEB expression and half maximal inhibitory concentration (IC50) of CDDP using Genomics of Drug Sensitivity in Cancer (GDSC) data. As shown in Fig. 2A, the expression level of TFEB was positively correlated with the sensitivity of OC cells to CDDP. Next, we compared TFEB expression in a panel of human OC cell lines (A2780, OV90, SKOV3, and OVCAR4 cells) and found that TFEB expression was positively correlated with the IC50 of CDDP (Fig. 2B and Suppl Fig. 1E). Furthermore, overexpression of TFEB significantly increased the tolerance of A2780 cells to CDDP (Fig. 2C, D). As is well known, TFEB activity depends on its nuclear translocation. Therefore, we further analyzed the effect of CDDP on TFEB nuclear translocation. We prepared A2780-CDDP, a CDDP-resistant cell line derived from A2780 (Fig. 2E), and found that compared to TFEB in parental cells, TFEB was present in its active form (decreased phosphorylation status) in A2780-CDDP cells (Fig. 2F). These results were further validated by transfection of a TFEB-GFP plasmid in 293T cells with CDDP treatment (Fig. 2G). We also found noticeable activation of TFEB in vivo with CDDP treatment (Fig. 2H). According to previous reports, TFEB-mediated lysosomal biogenesis confers resistance to chemotherapy drugs, including CDDP, in tongue squamous cell carcinoma^24^. In our study, we also found that TFEB depletion significantly suppressed lysosome biogenesis and their associated gene expression in resistant cells (Fig. 2I, J). Disrupting lysosomes with bafilomycin A1 increased the sensitivity of resistant cells to CDDP (Fig. 2K).

### TFEB mediates immune evasion by up-regulating the expression of PD-L1, PD-L2, and major histocompatibility complex class I in OC cells

**Fig. 3:**
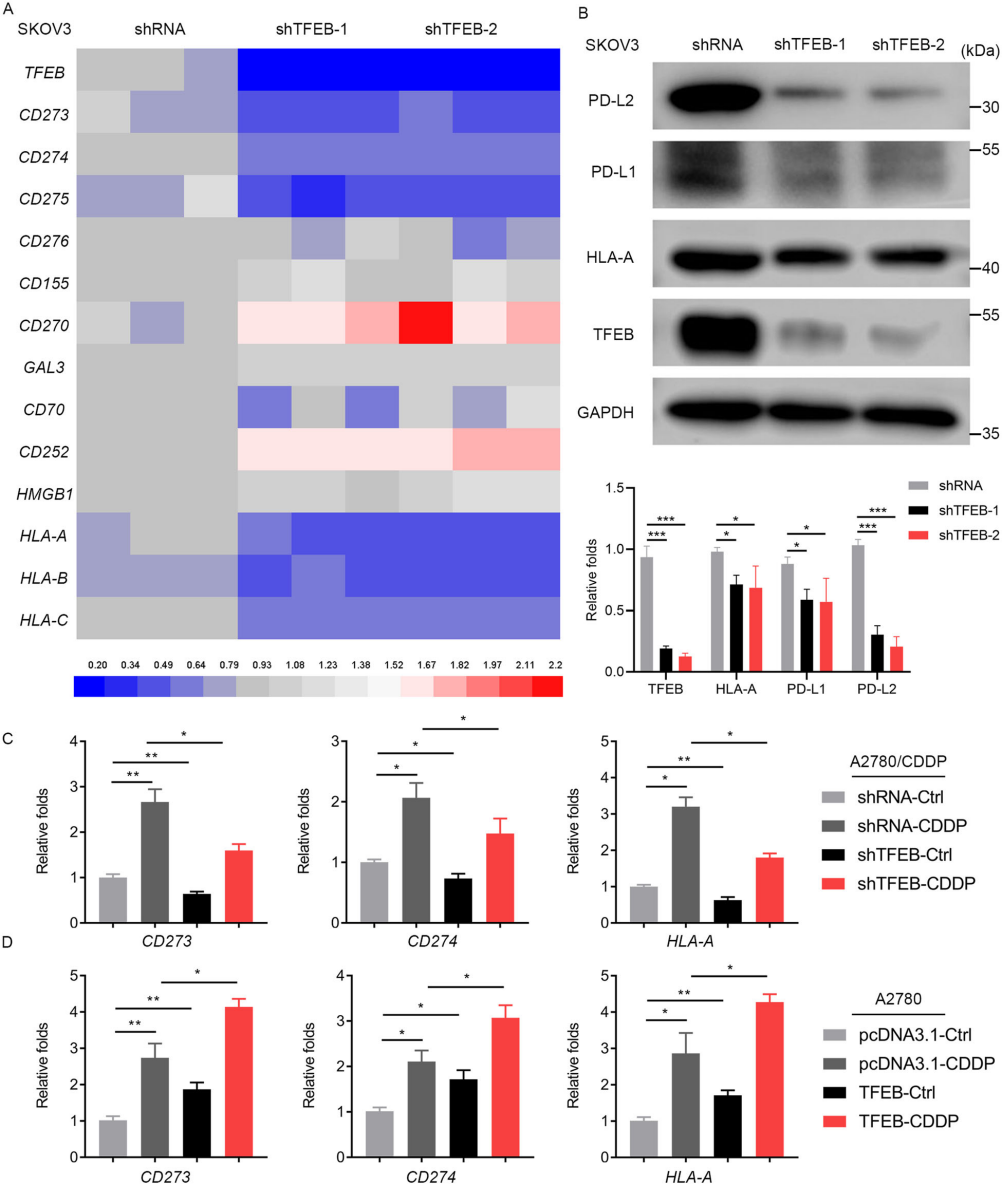
TFEB mediates immune evasion by up-regulating the expression of PD-L1, PD-L2, and MHC class I in OC cells. **A** The expression of immune checkpoint markers in SKOV3 cells was detected by qRT-PCR. SKOV3 cells were transduced with a scramble short hairpin RNA (shRNA) and TFEB shRNA (shTFEB-1, shTFEB-2) lentiviral particles. **B** The protein expression of TFEB, PD-L2, PD-L1, and HLA-A in SKOV3 cells was determined by western blotting. Representative histograms were shown. **C** *CD273*, *CD274*, and *HLA-A* expression in A2780-CDDP cells were determined by qRT-PCR. A2780-CDDP cells were transduced with shRNA or shTFEB lentiviral particles and were treated with or without CDDP (10 µM, 24 h). **D** *CD273*, *CD274*, and *HLA-A* expression in A2780 cells were determined by qRT-PCR. A2780 cells were transduced with a empty vector plasmid (pcDNA3.1) or a pcDNA3.1-TFEB plasmid (TFEB) and were treated with or without CDDP (10 µM, 24 h). Data are expressed as mean ± standard deviation. *p < 0.05; **p < 0.01, ***p < 0.001.

A recent study showed that TFEB can mediate immune evasion in RCC by up-regulating the expression of PD-L1^26^. Next, we explored whether TFEB can also mediate immune evasion by regulating the expression of immune checkpoint markers in OC. As shown in Fig. 3A, TFEB silencing downregulated the expression of some immune checkpoint markers such as *CD275* (ICOSL), *CD274* (PD-L1), *CD273* (PD-L2), and major histocompatibility complex (MHC) class I (*HLA-A*, *HLA-B*, and *HLA-C*), but induced the expression of other immune checkpoint markers such as *CD270* (HVEM) and *CD252* (TNFSF4). Given the important role of PD-L1, PD-L2, and MHC class I in regulating immune escape, we chose them for further study. To further clarify the effect of the regulation of TFEB on PD-L1, PD-L2, and HLA-A expression, we first downregulated TFEB in OC cells; we found that PD-L1, PD-L2, and HLA-A were downregulated accordingly (Fig. 3B). Next, we explored whether TFEB-driven changes in immune checkpoints were induced by CDDP. As shown in Fig. 3C, TFEB depletion inhibited *CD273*, *CD274*, and *HLA-A* transcription, and CDDP-induced elevations in *CD273*, *CD274*, and *HLA-A* levels were also significantly reduced in TFEB-deficient cells. In contrast, ectopic expression of TFEB induced *CD273*, *CD274*, and *HLA-A* expression and, in particular, facilitated *CD273, CD274*, and *HLA-A* transcription in the presence of CDDP (Fig. 3D). Together, these data demonstrate that CDDP can induce *CD273*, *CD274*, and *HLA-A* expression by activating TFEB in human OC cells.

### TFEB mediates immune evasion by up-regulating the expression of PD-L1, PD-L2, and MHC class I in OC patients

**Fig. 4:**
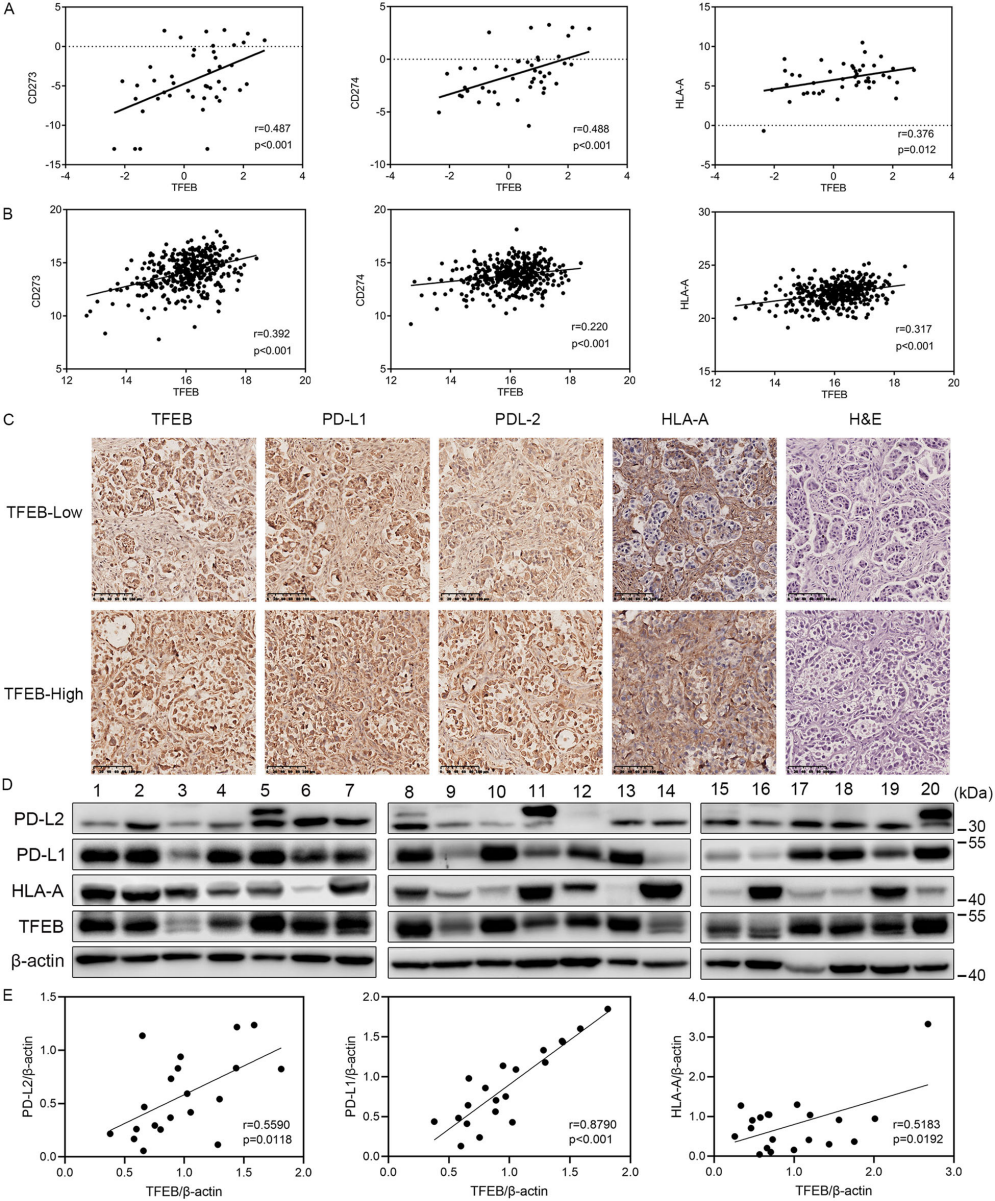
TFEB mediates immune evasion by up-regulating the expression of PD-L1, PD-L2, and MHC class I in OC patients. **A** The expression correlations of *TFEB*, *CD273*, *CD274*, and *HLA-A* in OC cell lines using the Broad Institute Cancer Cell Line Encyclopedia (CCLE). **B** The expression correlations of *TFEB*, *CD273*, *CD274*, and *HLA-A* in OC patients were observed by The Cancer Genome Atlas (TCGA) databases. **C** The expression of TFEB, PD-L1, PD-L2, and HLA-A was evaluated by immunohistochemistry analysis in 20 ovarian cancer patients. Scale bars = 100 μm. **D** The protein expression of TFEB, PD-L2, PD-L1, and HLA-A in 20 ovarian cancer patients was detected by western blotting. **E** The correlation of TFEB and PD-L2, PD-L1, and HLA-A expression in 20 ovarian cancer patients was plotted.

To further verify the positive correlation between TFEB and PD-L1, PD-L2, and HLA-A expression in OC cell lines, we analyzed publicly available data from the Broad Institute Cancer Cell Line Encyclopedia (CCLE) and TCGA databases on *TFEB*, *CD273*, *CD274*, *and HLA-A* expression. As shown in Fig. 4A, B, *TFEB* expression was positively correlated with *CD273*, *CD274*, *and HLA-A* expression in both OC cell lines and OC patients. We also assessed whether TFEB levels in patients with primary OC were related to the expression of PD-L1, PD-L2, and HLA-A. TFEB staining of human OC tissues showed heterogeneous expression, which can be readily differentiated into TFEB-Low and TFEB-High tissues (Suppl Fig. 1F). A higher expression of PD-L1, PD-L2, and HLA-A was observed in the TFEB-High tissues (Fig. 4C). These results were further confirmed by western blotting (Fig. 4D, E).

### Anti-PD-1 immunotherapy enhances the response to CDDP in OC

**Fig. 5:**
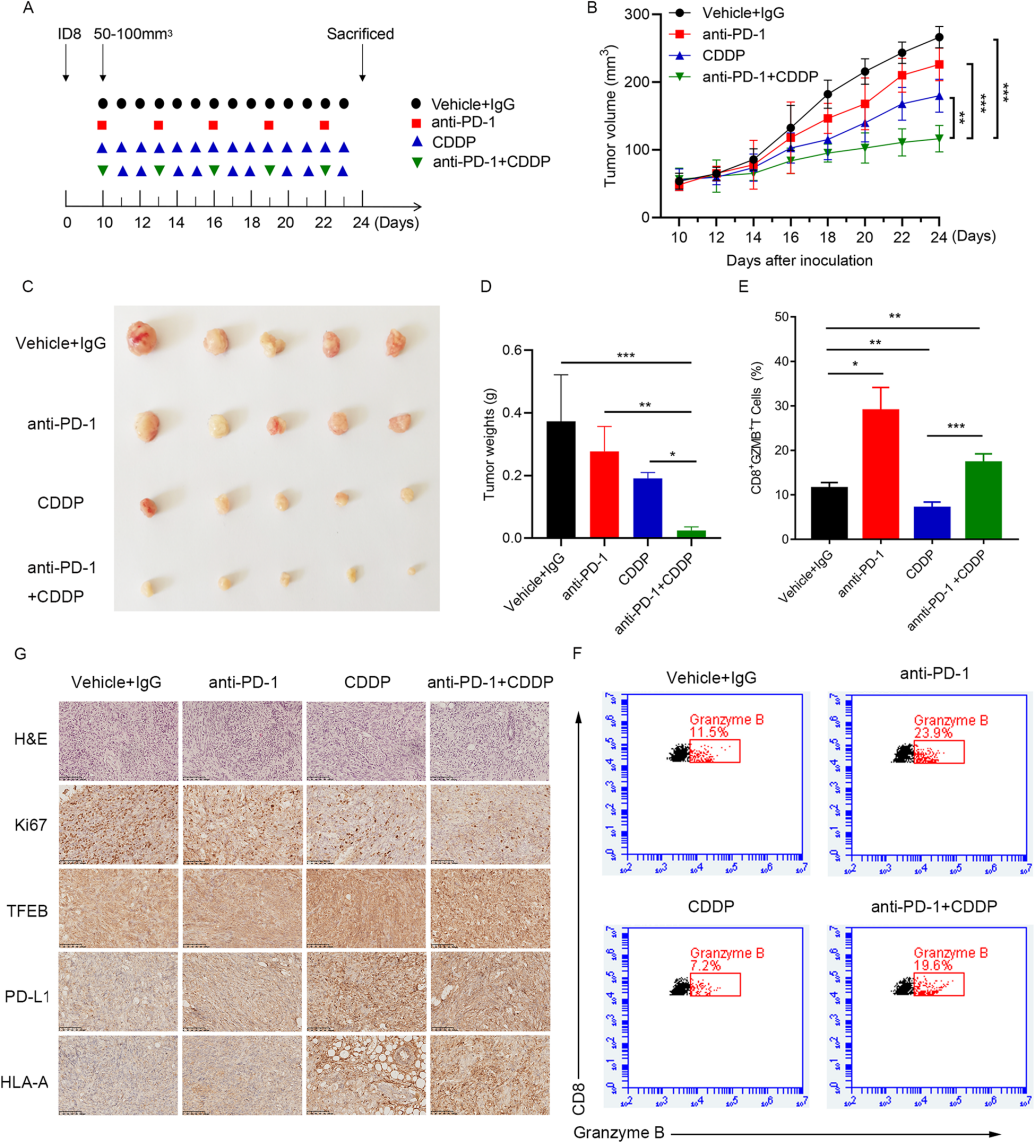
Anti-PD-1 immunotherapy enhances the response to CDDP in OC. **A** Schematic representation of the treatment is shown. **B** Comparison of tumor growth in different groups. **C** The mice were necropsied at day 24, and tumors are shown. **D** Comparison of tumor weights in different groups. **E** TILs were isolated and stained with CD8 and Granzyme B. Percentages of CD8^+^ Granzyme B ^+^ were shown. **F** TILs were isolated and stained with CD8 and Granzyme B. Representative scatter diagram were shown. **G** H&E and immunohistochemical staining of Ki67, TFEB, PD-L1, and HLA-A within tumor tissues. Scale bars = 100 μm. Data are expressed as mean ± standard deviation. *p < 0.05; **p < 0.01; ***p < 0.001.

Next, we determined whether the combined use of CDDP and anti-PD-1 antibody could enhance the effect of CDDP on OC growth in a syngenic murine model (Fig. 5A). Compared with the control group, tumor growth was reduced in mice treated with CDDP alone or anti-PD-1 alone (Fig. 5B–D). Furthermore, CDDP combined with anti-PD-1 treatment significantly reduced tumor size compared with the result of all the other groups (Fig. 5B–D). We also tested the effect of CDDP and PD-1 inhibition on cytotoxicity in tumor-infiltrating CD8^+^ T cells (CTL). CDDP treatment suppressed Granzyme B expression in CTL, but when combined with anti-PD-1, CDDP significantly enhanced their expression (Fig. 5E, F). As shown in Fig. 5G and Suppl Fig. 1G, CDDP enhanced the expression of TFEB and PD-L1 in tumor tissues, compared with the control group, indicating the significance of TFEB-PD-L1 axis in vivo during tumor growth. Ki67 immunohistochemical staining showed that anti-PD-1 alone had no inhibitory effect on tumor cell proliferation, while the inhibitory effect of CDDP on Ki67 staining was small but not significant, and CDDP combined with anti-PD-1 has a synergistic inhibitory effect on Ki67 staining (Fig. 5G). Taken together, these data demonstrate that the combined use of CDDP and anti-PD-1 may be a novel chemo-immunotherapeutic approach to treat OC.

## Discussion

This study explored whether TFEB is involved in remodeling the tumor immune microenvironment in OC. We found that TFEB did not affect cell proliferation but participated in CDDP resistance in OC. CDDP treatment induced TFEB nuclear translocation, thus increasing PD-L1 and PD-L2 expression to foster an immunosuppressive tumor microenvironment, which mediates immune evasion and drug resistance. TFEB also regulated HLA-A expression, which increases the tumor immunogenicity of OC. Finally, in the syngenic murine model of OC, we found a therapeutic effect of CDDP combined with anti-PD-1 antibody, which enhanced the cytolytic activity of CD8^+^ T cells and inhibited tumor growth.

Numerous studies have shown that MiT/TFE transcription factors play an important role in the maintenance of cellular physiological and pathological processes^15^. Out of the four members of the MiT/TFE family, the TFEB expression pattern is more common and its functions have been more extensively studied, which include metabolism, proliferation, autophagy, and immune responses^21^. TFEB expression is elevated in multiple types of human cancers, such as breast cancer, lung cancer, and pancreatic ductal adenocarcinoma. In these human cancers, amplification of TFEB expression is associated with multidrug resistance, aggressive behavior, and poor prognosis^27–30^. However, few studies have been conducted on OC. Only one transcriptome analysis showed that TFEB was involved in drug resistance in OC^31^, but the molecular mechanism remained unclear. In our study, we first found that downregulation of TFEB had no significant effect on cell proliferation in OC (Fig. 1). This finding is consistent with the latest report on RCC^26^. By further analysis, we found a significant positive correlation between the expression level of TFEB and the sensitivity of OC cells to CDDP (Fig. 2A, B and Suppl Fig. 1E). TFEB overexpression significantly increased the tolerance of A2780 cells to CDDP (Fig. 2C, D). Moreover, CDDP significantly induced TFEB nuclear translocation (Fig. 2F–H). As mentioned above, TFEB-mediated lysosomal biogenesis confers resistance to chemotherapy drugs, including CDDP^22–25^. In our study, we found that TFEB depletion significantly suppressed lysosomes and their associated gene expression in resistant cells, and disrupting lysosomes with bafilomycin A1 increased the sensitivity of resistant cells to CDDP (Fig. 2I–K). These findings indicate that TFEB-mediated lysosomal biogenesis induced by CDDP may be one of the potent tumor drug-resistance mechanisms in several cancers, including OCs.

Platinum- and paclitaxel-based chemotherapy are the most widely accepted standard of care for OC patients. However, they have long been known to cause systemic immunosuppressive effects due to bone marrow toxicity^32^. Nonetheless, recent studies have shown that chemotherapeutic drugs can alter the local immune state and affect the tumor responses to treatment^33,34^. In addition, a new form of apoptotic cell death, termed “immunogenic cell death”, releases damage-associated molecular patterns and elicits antigen-specific immune responses, which, in theory, synergize with immunotherapy^35,36^. Therefore, chemotherapeutic drugs may have dual effects (immunogenicity and immunosuppression) on the regulation of the immune microenvironment. Peng et al. reported that chemotherapy induces local immune suppression in OC through NF-kB-mediated PD-L1 upregulation^33^. Recently, Zhang et al.^26^ found that TFEB induces PD-L1 to mediate RCC immune evasion and resistance to mTOR inhibition. These findings led us to investigate whether the resistance to CDDP, the standard treatment for OC, is influenced by the upregulation of PD-L1 by TFEB in OC. Consequently, we found a positive correlation between TFEB and PD-L1, PD-L2, and HLA-A expression in OC cells and tissues (Figs. 3 and 4). CDDP treatment enhanced TFEB nuclear translocation and the expression of PD-L1, PD-L2, and HLA-A. A high expression of PD-L1 and PD-L2 is known to play an important role in mediating the immune escape of tumors, but the overexpression of MHC class I in tumor cells can increase the immunogenicity of the tumor and increase CTL infiltration at the tumor site^37^. Our study sheds light on the mechanism by which chemotherapeutic agents commonly used for OC enhance both the immunogenicity of tumor cells and the immunosuppressive tumor microenvironment at the tumor site via TFEB.

Immunotherapy with ICIs, especially anti-PD-1 and/or anti-PD-L1, has become a clinically effective treatment modality for various cancers and has provided unprecedented long-term survival in these cancers^7–9^. Immunotherapy is also considered promising for OC treatment. In an early study, the presence of CD3^+^ TILs were correlated with improved clinical outcomes^13^. A meta-analysis also demonstrated that CD8^+^ TILs were associated with a good survival advantage^14^. Later studies by Curiel et al.^38^ further demonstrated the prognostic significance of FOXP3^+^ T regulatory cells (Tregs) and/or the ratio of CTLs to FOXP3^+^ Tregs in OC^39^. Therefore, there is a solid rationale for the use of ICIs in patients with OC. CDDP has been proven to increase the permeability of tumor cell membranes to granzymes, thereby increasing the sensitivity of tumor cells towards lymphocyte killing^40^. Therefore, the combined use of CDDP and different cancer immunotherapy approaches could improve the efficacy of these treatments. Carboplatin/paclitaxel combined with PD-1 blockade has been successful in the treatment of non-small-cell lung cancer^41,42^. Combined immunochemotherapy with paclitaxel and anti-PD-1 antibody also showed a synergistic antitumor effect in a xenograft mouse model of OC^33^. In our study, we found that the combined use of CDDP and anti-PD-1 antibody enhanced the effect of CDDP on OC growth in our syngenic murine model. Thus, chemoimmunotherapy with CDDP and anti-PD-1 antibody may offer a novel and effective approach for treating OC.

In conclusion, we explored a novel function of TFEB in remodeling the tumor immune microenvironment in OC. Chemotherapy with CDDP induced several immunological changes in OC, including PD-L1, PD-L2, and MHC class I upregulation via TFEB signaling. Because of the important role of the TFEB/PD-L1 and MHC axis in regulating the tumor microenvironment with chemotherapy, TFEB is expected to become a good biomarker for immunotherapy. Chemoimmunotherapy with CDDP and anti-PD-1 antibody may offer a novel and effective approach to treatment for OC.

## Materials and methods

### Clinical specimens

Twenty ovarian carcinoma samples were collected at the Department of Gynecology of the Shandong Provincial Hospital Affiliated to Shandong First Medical University. None of the patients with OC received preoperative treatment. According to the ethical and legal standards, informed consent was obtained from all selected patients. This study was approved by the Ethics Committee of the hospital.

### Cell culture

OC cell lines A2780, SKOV3, OVCAR4, and OV90 were cultured in RPMI 1640 medium (Gibco, Rockville, MD, USA) supplemented with 1% penicillin–streptomycin (Macgene, Beijing, China) and 10% fetal bovine serum (FBS; Biological Industries, Kibbutz Beit HaEmek, Israel). ID8 cells were grown in high-glucose Dulbecco’s Modified Eagle Medium (DMEM) containing 1% penicillin–streptomycin solution and 10% FBS. The cells were incubated under 5% CO_2_ at 37 °C. To acquire CDDP-resistant cells, CDDP (MedChemExpress, Monmouth Junction, NJ, USA) was added to the medium at gradually increasing concentrations from 5 µM to 20 µM. During the continuous exposure, the CDDP medium was replaced with fresh medium containing the specified concentration of CDDP every 3 days and maintained at this concentration for at least four generations. Cells that survived at concentrations up to 20 µM were named A2780/CDDP cells.

### Transfection

Lentiviral shRNA interference vectors targeting TFEB (shTFEB-1 and shTFEB-2) and negative control (short hairpin RNA [shRNA]) were purchased from Hanbio Biotechnology (Shanghai, China). The TFEB shRNAs were as follows: shTFEB-1 (forward), 5′-CCGGCCCACTTTGGTGCTAATAGCTCTCGAGAGCTATTAGCAC

CAAAGTGGGTTTTTG-3′; shTFEB-1 (reverse), 5′-AATTCAAAAACCCACTTTGG

TGCTAATAGCTCTCGAGAGCTATTAGCACCAAAGTGGG-3′; shTFEB-2 (forward), 5′-CCGGCGATGTCCTTGGCTACATCAACTCGAGTTGATGTAGCCA

AGGACATCGTTTTTG-3′; and shTFEB-2 (reverse), 5′-AATTCAAAAACGATGTC

CTTGGCTACATCAACTCGAGTTGATGTAGCCAAGGACATCG-3′. The pcDNA3.1-TFEB plasmid and negative control (pcDNA3.1) used for cell transfection were synthesized by GenePharma (Shanghai, China). Transfections were performed with Lipofectamine^TM^ 3000 (Invitrogen, Carlsbad, CA, USA) according to the manufacturer’s protocol.

### qRT-PCR

Total RNA from cells was extracted using TRIzol Reagent and 1 µg of the RNA was reverse transcribed to cDNA. Real-time quantitative reverse transcription polymerase chain reaction (qRT-PCR) was performed using the SYBR® Premix Ex Taq™ II (Takara, Shiga, Japan) and the Light Roche 480 System. The comparative expression level of genes was compared with that of *β-actin* using the 2^−ΔΔCt^ method. The primers used in this experiment are listed in Table 1.

**Table 1:** Primers.

Name (human)	Forward primer	Reverse primer
*β-ACTIN*	CATGTACGTTGCTATCCAGGC	CTCCTTAATGTCACGCACGAT
*GAPDH*	GGAGCGAGATCCCTCCAAAAT	GCTGTTGTCATACTTCTCATG
*TFEB*	CCAGAAGCGAGAGCTCACAGAT	TGTGATTGTCTTTCTTCTGCCG
*LAMP1*	ACGTTACAGCGTCCAGCTCAT	TCTTTGGAGCTCGCATTGG
*LAMP2*	TGGCAATGATACTTGTCTGCTG	ACGGAGCCATTAACCAAATACAT
*CTSB*	AGTGGAGAATGGCACACCCTA	AAGAAGCCATTGTCACCCCA
*CTSL*	CTTTTGCCTGGGAATTGCCTC	CATCGCCTTCCACTTGGTC
*CD273*	ACCAGTGTTCTGCGCCTAA	CCTGGGTTCCATCTGACTTTG
*CD274*	GGTAAGACCACCACCACCAAT	TGATTCTCAGTGTGCTGGTCAC
*CD275*	CGTCTTCTTGAACATGCGGG	TTTTCTCGCCGGTACTGACT
*CD276*	CTCACAGGAAGATGCTGCGT	CTGTGAGGCAGAACCACAGT
*CD155*	AGGCTATAATTGGAGCACGACC	GGTTTGTCCACAGGACGGAT
*CD270*	CAAGGTGATCGTCTCCGTCC	TCTGTGGGTCAGTGGTTTGG
*GAL3*	ATAACCTGCCTTTGCCTGGG	AGCAATTCTGTTTGCATTGGGC
*CD70*	GTCACTTGGGTGGGACGTA	CAGTATAGCCTGGGGTCCTG
*CD252*	GAGCCCCTCTTCCAACTGAA	CAGTTCTCCGCCATTCACAT
*HMGB1*	TATGGCAAAAGCGGACAAGG	CTTCGCAACATCACCAATGGA
*HLA-A*	ATACCTGGAGAACGGGAAGGAG	GAGATGGGGTGGTGGGTCATA
*HLA-B*	CAGTTCGTGAGGTTCGACAG	CAGCCGTACATGCTCTGGA
*HLA-C*	CCATGAGGTATTTGTGGACCG	TCTCGGACTCTCGTCGTCG

### Western blotting

Proteins were extracted with radioimmunoprecipitation assay (RIPA) lysis buffer and boiled for denaturation. Sodium dodecyl sulphate–polyacrylamide gel electrophoresis (SDS-PAGE) was performed on samples containing 30 µg of protein using a 10% polyacrylamide gel. After electrotransfer and blocking with 3% bovine serum albumin, the membranes were incubated overnight at 4 °C with primary antibodies. Rabbit antibodies against TFEB, HLA-A, GAPDH, Histone-H3 (1:2000; Proteintech, China), PD-L1, PD-L2 (1:5000; Abcam, USA), and mouse antibody against β-actin (1:5000; Proteintech) were used as primary antibodies. After incubating with anti-rabbit or anti-mouse secondary antibody (1:5000; Proteintech) for 60 min at room temperature, enhanced chemiluminescence (ECL, NJ, USA) was used to visualize the bands.

### Colony formation assay

Cells were seeded in six-well plates at 500 cells per well. After incubating in 5% CO_2_ at 37 °C for 10 days, cells were fixed with 4% paraformaldehyde for 30 min and stained with 0.1% crystal violet at room temperature for 10 min. Colonies containing at least 50 cells were counted under the microscope.

### CCK-8 assay

The cells were seeded onto a 96-well plate at 10,000 cells per well. The cells were incubated for 12, 24, 36, 48 h, and 10 µl of Cell Counting Kit-8 solution (Dojindo, Kumamoto, Japan) was added to each well. After 1.5 h of incubation at 37 °C, the absorbance at 450 nm was measured using a microplate reader (EL340, Bio-Tek Instruments, MA, USA).

### xCELLigence

Experiments were carried out using the RTCADP instrument (Roche, Germany). In all, 10,000 cells/well were added to 16 well E-Plates. The electronic sensors provided a continuous and quantitative measurement of cell index in each well.

### Cell apoptosis assay

After digestion in the six-well plates, the cells were washed twice with phosphate-buffered saline (PBS) solution. Cells were centrifuged at 1500 rpm for 5 min to collect cells and then stained with 7-AAD and PE Annexin V (BD Biosciences, Hercules, CA, USA) for 15 min in room temperature. The apoptotic cells were measured using flow cytometry (BD Biosciences).

### Nuclear and cytoplasmic extraction

The PARIS™ kit (AM1556; Thermo Fisher Scientific, Waltham, USA) was used to separate the cytoplasm and nucleus according to the manufacturer’s instructions. Briefly, cells were lysed in cell fraction buffer for 10 min on ice, centrifuged at 500×*g* at 4 °C for 3 min, and the supernatant was collected as the cytoplasmic fraction. The pellet was then washed with cell debris buffer to collect nuclei. Then nuclear and cytoplasmic proteins were extracted for western blotting.

### Fluorescence microscopy

293T cells transfected with a TFEB-GFP plasmid were seeded on glass slides and cultured overnight for proper attachment. Cells were stimulated with vehicle or CDDP (10 µM) for 12 h, washed three times with PBS, and fixed with 4% paraformaldehyde for 10 min. Next, slides were incubated in DAPI for 10 min to stain the nuclei. The samples were then imaged using a confocal microscope (Leica; Germany).

### Lyso-Tracker Red Staining

Lysosomal staining was performed using Lyso-Tracker Red (LTR). After cells were stably adherent to the wall in a 20-mm glass bottom dish, cells (1.0 × 10^6^ cells/ml) were incubated with Lyso-Tracker Red at 5 nmol/L for 30 min at 37 °C and washed three times with phosphate-buffered saline (PBS). Next, slides were incubated in DAPI for 10 min to stain the nuclei. The cells were then inspected and photographed with a confocal microscope.

### Hematoxylin and eosin staining

Fresh OC tissues were excised and fixed in paraformaldehyde. After conventional dehydration and paraffin-embedding, slices were prepared, dewaxed with xylene, dehydrated with gradient alcohol, and then stained with hematoxylin for 4 min followed by eosin for 1 min. The slices were observed under an Olympus light microscope.

### Immunohistochemistry

Immunohistochemical analysis was performed according to the manufacturer’s instructions. Briefly, the slices were incubated with primary antibodies against TFEB, PD-L1, PD-L2, HLA-A (Proteintech), and Ki67 (Abcam) at 4 °C overnight. Next, slides were incubated with secondary antibodies at room temperature for 2 h. The mean staining intensity (MSI) of TFEB in human OC tissues was analyzed by TissueFAXS Plus (TissueGnostics). The 20 cases were equally divided into the TFEB-Low group and TFEB-High group according to the MSI. The correlation between the TFEB level of patients with primary OC and the expression of PD-L1, PD-L2, and HLA-A was evaluated.

### Syngenic murine model of ovarian cancer

C57BL/6 female mice were injected subcutaneously with 5 × 10^6^ ID8 cells in 0.1 ml of PBS. When the tumor volume reached 50 mm^3^, mice were treated with either CDDP (2.5 mg/kg, intraperitoneally [i.p.]) daily, anti-PD-1 (200 μg/mouse, i.p.) (BE0101, BioXCell, Lebanon, NH, USA) five times in 12 days, a combination of both, or a vehicle plus control IgG. Tumor volumes were measured along the major axis (a) and minor axis (b) every second day and calculated using the formula *V* = (a × b^2^)/2. After 2 weeks, the mice were euthanized and the tumors were dissected and weighed.

### Isolation of primary tumor cells and TILs

Tumor specimens were gently minced into small pieces and digested with 3 ml PBS containing 1 mg/ml collagenase IV (17104019; Invitrogen, Carlsbad, CA, USA) for 30 min at 37 °C. Cell suspensions were filtered twice and centrifuged at 1500 rpm for 5 min. Cell precipitates were resuspended in PBS, and tumor cells and tumor-infiltrating lymphocytes (TILs) were collected.

### Flow cytometry

The antibodies used to stain TILs were as follows: FITC anti-mouse CD3 (100203; BioLegend, CA, USA), PE anti-mouse CD4 (100407; BioLegend), Percp/Cy5.5 anti-mouse CD8a (100733; BioLegend), and APC anti-mouse Granzyme B (372203; BioLegend). Using BD LSRFortessa flow cytometer to collect samples, and using FlowJo software for data analysis.

### Statistical analysis

Statistical analysis was performed using GraphPad Prism 8.0.2 software. The data of three independent repeated experiments are expressed as mean ± standard deviation. Statistical significance was determined using Student’s *t* test or one-way analysis of variance (ANOVA). *p*-values of <0.05 were considered significant.

## Supplementary information

Supplementary figure 1

Supplementary information

## Data Availability

The datasets used and/or analyzed during the study are available from the corresponding author upon reasonable request.

## References

[1] Bray F (2018). Global cancer statistics 2018: GLOBOCAN estimates of incidence and mortality worldwide for 36 cancers in 185 countries. CA A Cancer J. Clin..

[2] Bergamini A, Bocciolone L, Fodor A, Candiani M, Mangili G (2019). Management of recurrent ovarian cancer: when platinum-based regimens are not a therapeutic option. Int. J. Gynecol. Cancer.

[3] Nowak M. & Klink M. The role of tumor-associated macrophages in the progression and chemoresistance of ovarian cancer. *Cells* **9**, 1299 (2020) 10.3390/cells9051299 PMC7290435 32456078

[4] Larionova I (2019). Interaction of tumor-associated macrophages and cancer chemotherapy. Oncoimmunology.

[5] Peng J (2015). Chemotherapy induces programmed cell death-ligand 1 overexpression via the nuclear factor- B to foster an immunosuppressive tumor microenvironment in ovarian cancer. Cancer Res..

[6] Cavazzoni, A. et al. Pemetrexed enhances membrane PD-L1 expression and potentiates T cell-mediated cytotoxicity by anti-PD-L1 antibody therapy in non-small-cell lung cancer. *Cancers* **12**, 666 (2020) 10.3390/cancers12030666 PMC7139811 32178474

[7] Kotsakis A, Georgoulias V (2017). Avelumab, an anti-PD-L1 monoclonal antibody, shows activity in various tumour types. Lancet Oncol..

[8] Hamid O (2013). Safety and tumor responses with lambrolizumab (anti-PD-1) in melanoma. N. Engl. J. Med..

[9] Topalian SL (2012). Safety, activity, and immune correlates of anti-PD-1 antibody in cancer. N. Engl. J. Med..

[10] Cheng S (2020). Artificial mini dendritic cells boost T cell-based immunotherapy for ovarian cancer. Adv. Sci..

[11] Le Saux O (2020). [Current advances in immunotherapy in ovarian cancer]. Bull. Cancer.

[12] Brewer M (2020). Front-line chemo-immunotherapy with carboplatin-paclitaxel using oregovomab indirect immunization in advanced ovarian cancer: a randomized phase II study. Gynecol. Oncol..

[13] Zhang L (2003). Intratumoral T cells, recurrence, and survival in epithelial ovarian cancer. N. Engl. J. Med..

[14] Hwang WT, Adams SF, Tahirovic E, Hagemann IS, Coukos G (2012). Prognostic significance of tumor-infiltrating T cells in ovarian cancer: a meta-analysis. Gynecol. Oncol..

[15] Yang M (2018). Emerging roles and regulation of MiT/TFE transcriptional factors. Cell Commun. Signal..

[16] Brady OA, Martina JA, Puertollano R (2018). Emerging roles for TFEB in the immune response and inflammation. Autophagy.

[17] Raben N, Puertollano R (2016). TFEB and TFE3: linking lysosomes to cellular adaptation to stress. Annu. Rev. Cell Dev. Biol..

[18] Kauffman EC (2014). Molecular genetics and cellular features of TFE3 and TFEB fusion kidney cancers. Nat. Rev. Urol..

[19] Zhang W, Li X, Wang S, Chen Y, Liu H (2020). Regulation of TFEB activity and its potential as a therapeutic target against kidney diseases. Cell Death Discov..

[20] Sakamoto H (2018). Transcription factor EB influences invasion and migration in oral squamous cell carcinomas. Oral Dis..

[21] Bahrami A, Bianconi V, Pirro M, Orafai HM, Sahebkar A (2020). The role of TFEB in tumor cell autophagy: Diagnostic and therapeutic opportunities. Life Sci..

[22] Zhao B (2020). TFEB-mediated lysosomal biogenesis and lysosomal drug sequestration confer resistance to MEK inhibition in pancreatic cancer. Cell Death Discov..

[23] Slade L (2020). A lysosome independent role for TFEB in activating DNA repair and inhibiting apoptosis in breast cancer cells. Biochem. J..

[24] Chu HY (2018). Bafilomycin A1 increases the sensitivity of tongue squamous cell carcinoma cells to cisplatin by inhibiting the lysosomal uptake of platinum ions but not autophagy. Cancer Lett..

[25] Karagounis IV (2016). Repression of the autophagic response sensitises lung cancer cells to radiation and chemotherapy. Br. J. Cancer.

[26] Zhang C (2019). TFEB mediates immune evasion and resistance to mTOR inhibition of renal cell carcinoma via induction of PD-L1. Clin. Cancer Res..

[27] Perera RM (2015). Transcriptional control of autophagy-lysosome function drives pancreatic cancer metabolism. Nature.

[28] Giatromanolaki A (2015). Increased expression of transcription factor EB (TFEB) is associated with autophagy, migratory phenotype and poor prognosis in non-small cell lung cancer. Lung Cancer.

[29] Giatromanolaki A, Sivridis E, Kalamida D, Koukourakis MI (2017). Transcription factor EB expression in early breast cancer relates to lysosomal/autophagosomal markers and prognosis. Clin. Breast Cancer.

[30] Klein K (2016). Role of TFEB-driven autophagy regulation in pancreatic cancer treatment. Int. J. Oncol..

[31] Kim YR (2015). Transcriptome analysis indicates TFEB1 and YEATS4 as regulatory transcription factors for drug resistance of ovarian cancer. Oncotarget.

[32] Nowak AK, Robinson BW, Lake RA (2003). Synergy between chemotherapy and immunotherapy in the treatment of established murine solid tumors. Cancer Res..

[33] Peng J (2015). Chemotherapy induces programmed cell death-ligand 1 overexpression via the nuclear factor-kappab to foster an immunosuppressive tumor microenvironment in ovarian cancer. Cancer Res..

[34] Yang M (2017). Chemotherapy induces tumor immune evasion by upregulation of programmed cell death ligand 1 expression in bone marrow stromal cells. Mol. Oncol..

[35] Asadzadeh, Z. et al. Current approaches for combination therapy of cancer: the role of immunogenic cell death. *Cancers* **12**, 1047 (2020) 10.3390/cancers12041047 PMC7226590 32340275

[36] Zhou J (2019). Immunogenic cell death in cancer therapy: Present and emerging inducers. J. Cell. Mol. Med..

[37] Mortezaee K (2020). Immune escape: a critical hallmark in solid tumors. Life Sci..

[38] Curiel TJ (2004). Specific recruitment of regulatory T cells in ovarian carcinoma fosters immune privilege and predicts reduced survival. Nat. Med..

[39] Jackson SR, Yuan J, Teague RM (2014). Targeting CD8+ T-cell tolerance for cancer immunotherapy. Immunotherapy.

[40] Ramakrishnan R (2010). Chemotherapy enhances tumor cell susceptibility to CTL-mediated killing during cancer immunotherapy in mice. J. Clin. Investig..

[41] Kroon P (2019). Radiotherapy and cisplatin increase immunotherapy efficacy by enabling local and systemic intratumoral T-cell activity. Cancer Immunol. Res..

[42] Lazzari C (2018). Combination of immunotherapy with chemotherapy and radiotherapy in lung cancer: is this the beginning of the end for cancer?. Ther. Adv. Med. Oncol..

