# Supplementary figures and images for: Cisplatin remodels the tumor immune microenvironment via the transcription factor EB in ovarian cancer
Source: Cell Death Discov. 2021 Jun 5;7:136. doi: 10.1038/s41420-021-00519-8 (PMC8179924; doi:10.1038/s41420-021-00519-8)

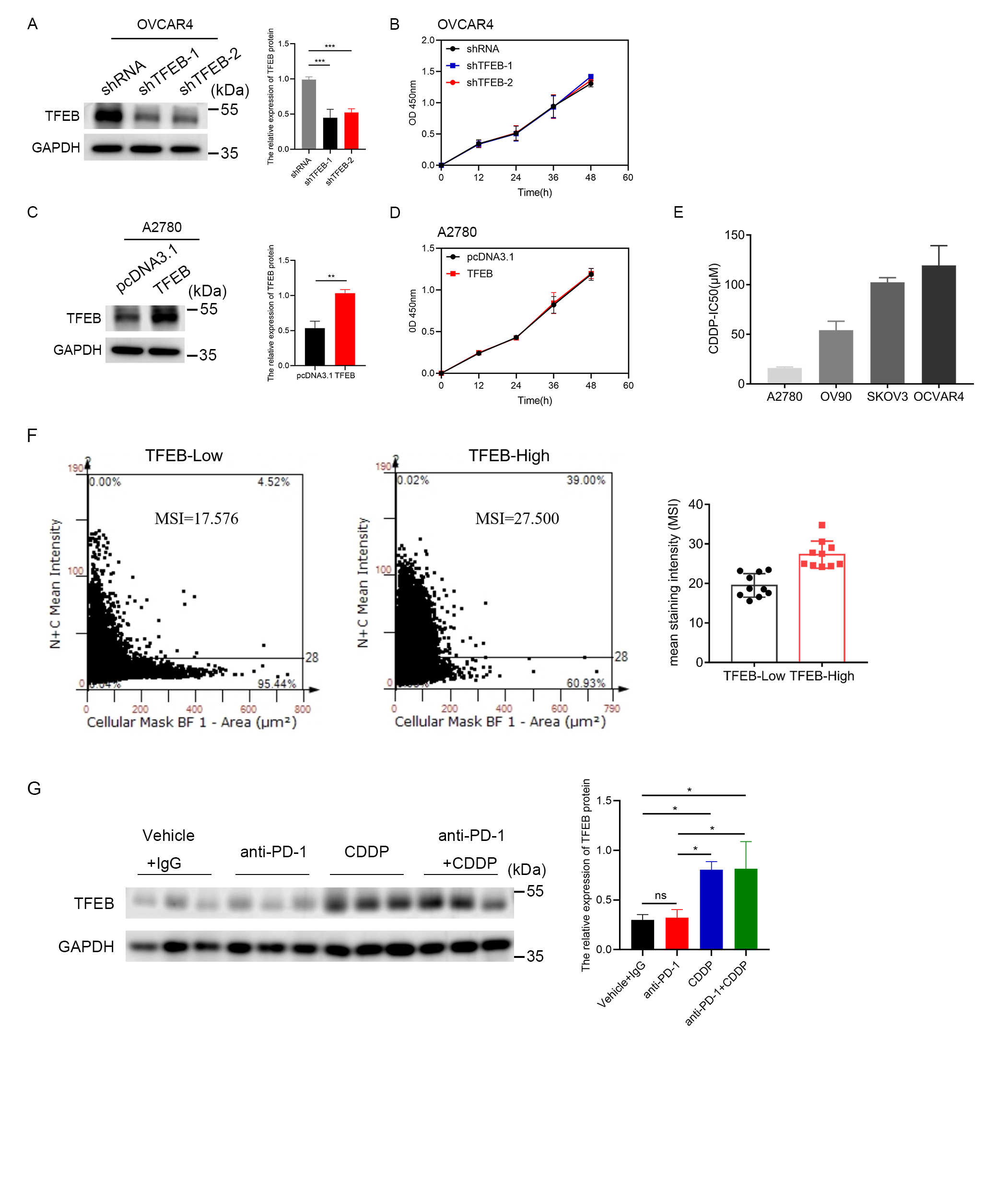

Supplement: Supplementary file 1 — Supplementary figure 1 [file 41420_2021_519_MOESM1_ESM.tif]
