# Supplementary material for: Cisplatin remodels the tumor immune microenvironment via the transcription factor EB in ovarian cancer
Source: Cell Death Discov. 2021 Jun 5;7:136. doi: 10.1038/s41420-021-00519-8 (PMC8179924; doi:10.1038/s41420-021-00519-8)
Supplement: Supplementary file 2 — Supplementary information [file 41420_2021_519_MOESM2_ESM.docx]

**Supplementary information**

**Supplementary Table. 1 Primer sequences in this study.**

**Supplementary Fig. 1 Effects of TFEB on proliferation of OC cells, and TFEB expression in human OC tissues and syngenic** **murine model of OC.** (A) OVCAR4 cells were transduced by scramble shRNA (shRNA) and TFEB shRNA (shTFEB-1, shTFEB-2) lentiviral particles. The expression of TFEB was detected by western blotting and the significance of the change of band intensities was detected by one-way analysis of variance. (B) Down-regulation of TFEB expression does not affect OVCAR4 cell growth, as detected by CCK-8 assays. (C) A2780 cells were transfected with either an empty vector plasmids (pcDNA3.1) or pcDNA3.1-TFEB plasmids (TFEB) and the expression of TFEB was determined by western blotting. (D) Up-regulation of TFEB expression does not affect A2780 cell growth, as detected by CCK-8 assays. (E) The IC50 of cell lines (A2780, OV90, SKOV3, and OVCAR4 cells) were detected by CCK-8 assays. (F) The mean staining intensity (MSI) of TFEB expression in 20 ovarian cancer tissues was analyzed by TissueFAXS Plus and divided equally according to the MSI. (G) The expression of TFEB in the mouse tumor tissues was detected by western blotting and the significance of the change of band intensities was detected by one-way analysis of variance. Data are expressed as mean±standard deviation. **p*<0.05; ***p*<0.01; ****p*<0.001.
